# Child Maltreatment Characteristics and Adult Physical Multimorbidity in Germany

**DOI:** 10.1001/jamanetworkopen.2024.56050

**Published:** 2025-01-23

**Authors:** Daniëlle Otten, Inga Schalinski, Jörg M. Fegert, Andreas Jud, Elmar Brähler, David Bürgin, Vera Clemens

**Affiliations:** 1 Department of Child and Adolescent Psychiatry-Psychotherapy, University Hospital Ulm, Ulm, Germany; 2 Department of Psychosomatic Medicine and Psychotherapy, University Medical Center, Johannes Gutenberg-University Mainz, Mainz, Germany; 3 German Center for Mental Health, Partner Site Ulm, Germany; 4 German Center for Child and Adolescent Health, Partner Site Ulm, Germany; 5 Universität der Bundeswehr München, Department of Human Sciences, Institute of Psychology, Munich, Germany; 6 Department of Medical Psychology and Medical Sociology, University of Leipzig, Leipzig, Germany; 7 Child and Adolescent Psychiatric Research Department, University Psychiatric Clinics Basel, University of Basel, Basel, Switzerland; 8 Jacobs Center for Productive Youth Development, University of Zurich, Zurich, Switzerland

## Abstract

**Importance:**

Associations between child maltreatment (CM) and health have been studied broadly, but most studies focus on multiplicity (number of experienced subtypes of CM). Studies assessing multiple CM characteristics are scarce, partly due to methodological challenges, and were mostly conducted in patient samples.

**Objective:**

To determine the importance of CM characteristics in association with physical multimorbidity in adulthood for women and men in a German representative sample.

**Design, Setting, and Participants:**

This survey study used data from a representative sample of the German population between July and October 2021. Households were randomly selected within regional areas by a German demographic consultation company, and the Kish-Grid method was applied to ensure random participation. Reasons for nonparticipation were refusal of the selected household or target person to participate and failure to contact a household after 4 visits. Analyses took place between June 2023 and July 2024.

**Exposure:**

Characteristics of CM, including subtypes of CM, multiplicity, age at time of maltreatment (timing), number of years of experienced maltreatment (duration), frequency, and subjective severity (measured with the ISPCAN Child Abuse Screening Tools Retrospective version questionnaire).

**Main Outcomes and Measures:**

The main outcome was physical multimorbidity, defined as the sum score of lifetime leading morbidity and mortality causes in Western countries (obesity, diabetes, cancer, hypertension, myocardial infarction, chronic obstructive pulmonary disease, and incident stroke). Conditioned random forest regression analyses (a machine learning regression technique) were conducted to examine what characteristics of CM were most importantly associated with physical multimorbidity in adulthood while considering all other variables in the model.

**Results:**

Of 5908 individuals invited, the study sample included 2514 participants (response rate, 42.6%), with 1297 (51.6%) women (mean [SD] age, 50.6 [17.9] years) and 1217 (48.4%) men (mean [SD] age, 49.5 [18.2] years). Duration of CM was the most important factor for physical multimorbidity in adulthood for both women (importance = 0.595; 95% CI, 0.599-0.601) and men (importance = 1.389; 95% CI, 1.386-1.394). Duration and timing variables were more importantly associated with outcomes than multiplicity in women and men. For women, severity and experiencing CM at age 4 years was significantly associated with physical multimorbidity in adulthood. For men, experiencing CM at age 11 years was most importantly associated with physical multimorbidity in adulthood.

**Conclusions and Relevance:**

In this survey study, conditioned random forest regression analyses were applied to provide insights in the importance of duration and timing of CM for physical health in addition to the frequently studied multiplicity. These findings suggest that CM assessments should be considered in diagnostics of individuals with physical health conditions and may also inform strategies to mitigate the risk.

## Key Points

**Question:**

What characteristics of child maltreatment (CM) have the most important associations with physical multimorbidity in adulthood?

**Findings:**

In this survey study including 2514 respondents in Germany, duration of CM (number of years of exposure) was associated with physical multimorbidity in adulthood for women and men. Time of exposure was important, with 4 years being significant for women and 11 years for men; both the duration of exposure and the specific timing were more important than multiplicity of CM.

**Meaning:**

These findings suggest that CM assessments, including duration and timing, should be considered in diagnostics of individuals with multiple adverse physical health conditions.

## Teaser

This survey study examines the importance of child maltreatment characteristics in association with physical multimorbidity in adulthood for women and men in a German representative sample.

## Introduction

Child maltreatment (CM) refers to any intentional harm or threat of harm inflicted on a child by that child’s caregivers or other responsible party and includes physical, emotional, or sexual abuse and neglect (ie, acts of omission by parent or caregiver^1^). In Germany, prevalence of CM varies between 2.3% for severe sexual abuse and 9% for severe neglect.^2^

CM substantially contributes to child morbidity and mortality^3^ and can have devastating consequences for a person’s health in adulthood. Meta-analyses found multiple types of CM to be significant risk factors associated with cardiovascular, respiratory, neurological, liver or digestive, and metabolic conditions in adulthood,^4,5^ and these findings were also confirmed for Germany.^6,7^ These associations are often explained by several physiological pathways, such as profound alterations in the biological system related to metabolism and the immune system.^8^ Associations between CM and adverse health outcomes tend to differ between women and men. CM has been associated with adult obesity in men,^9^ whereas associations between CM and cancer are especially present in women.^10^

The presence of multiple physical diseases (multimorbidity) causes numerous adverse health outcomes resulting in excess mortality.^11,12^ Physical multimorbidity increases substantially with age^13^ (confirmed for the German population^14^). Although age dependent, cardiovascular and metabolic conditions often form the starting point of the accumulation of diseases resulting in multimorbidity,^15^ especially for men,^16^ whereas among older women, mechanical patterns (eg, cervical pain, osteoporosis) are more common.^16^

While studies to date mainly use the number of experienced subtypes of CM for estimation of associations with health status,^6,7^ this approach is somewhat controversial. Although it offers the possibility of identifying important risk indicators across general and clinical populations, it does not accurately identify individual risks of later health problems.^17,18^ Since CM is a complex heterogeneous problem, the nature of the maltreatment experiences and its timing and duration (ie, experiencing CM over a longer period of time or during different development periods) may have differential effects on health outcomes, as was shown for several mental health disorders^19^ (eg, shutdown dissociation,^20^ dissociative symptoms, posttraumatic stress disorder, and depression in patient samples^21,22^ and in the general population^23^). Other studies found systematic variations of type and timing of CM for neurobiological associations.^24,25,26^ Focusing merely on one aspect of maltreatment or creating a risk score by adding the number of distinct adversities experienced implicitly assumes that very different experiences influence health in similar ways, which is unlikely.

Therefore, it is important that studies focusing on associations between CM and somatic health over the life course assess the importance of timing of CM. Occurrence of CM during sensitive periods in development when the brain is maximally sensitive to particular types of environmental input^26^ could account for differential physical health outcomes in adulthood.

In this study, we aim to assess the association of CM characteristics, such as subtypes (ie, neglect and physical, emotional, and sexual abuse), timing (age at time of maltreatment), duration (number of years of experienced maltreatment), frequency, and subjective severity with physical multimorbidity (including overweight, diabetes, cancer, hypertension, myocardial infarction, chronic obstructive pulmonary disease [COPD], and incident stroke) in adulthood and aim to identify patterns for women and men using data from a large representative, population-based random sample.

## Methods

This survey study was conducted in accordance with the Declaration of Helsinki and fulfilled the ethical guidelines of the International Code of Marketing and Social Research Practice of the International Chamber of Commerce and of the European Society of Opinion and Marketing Research. The study was approved by the ethics committee of the medical department of the University of Leipzig. This study is reported following the American Association for Public Opinion Research (AAPOR) reporting guideline.

### Sample

A representative sample of the German population was generated by a demographic consultation company (USUMA). Data collection took place between July and October 2021. The Federal Republic of Germany was systematically divided into regional areas and divided into 128 networks that were used as the sampling frame. Each network contained 258 sample points, proportionate to the distribution of private households in Germany. Within each sample point, households were randomly selected. The Kish-Grid method was applied to ensure random participation. Inclusion criteria were age 16 years or older and sufficient German language understanding to participate. Individuals were given information about the study and provided informed consent. Face-to-face interview-format and written self-report questionnaires were used to assess demographic characteristics and information on somatic health.

Among 5908 individuals contacted, the final sample included 2515 individuals (response rate, 42.6%). Main reasons for nonparticipation were refusal of the selected household or target person to participate and failure to contact a household after 4 visits. In this study, persons identifying as either women or men were included, since we have no further information on persons indicating gender diverse.

### Measures

#### CM

CM was measured with the ISPCAN Child Abuse Screening Tools Retrospective version questionnaire.^27,28^ This tool, designed for young adults, retrospectively screens for neglect and physical, emotional, and sexual abuse before the age 18 years with follow-up questions on age at time of experience, frequency, and severity for those who experienced CM. Validity of the ISPCAN Child Abuse Screening Tools Retrospective version has been shown in a national German sample^29^ and revealed adequate to good internal consistency based on McDonald ω.

#### Subtypes of CM and Multiplicity

For each subtype of CM (neglect and physical, emotional, and sexual abuse), 5 acts were described. Individuals could confirm or affirm exposure to these acts or refrain from answering. Affirmation of exposure to at least 1 act was coded as having experienced the corresponding subtype of CM. A continuous variable for multiplicity (ie, exposure to multiple subtypes of CM) was calculated by summing the different subtypes of CM leading to a multiplicity score ranging from 0 to 4.

#### Timing and Duration

Timing of CM was measured with variables indicating for each year of age the exposure to any type of CM (coded 1). Years of age without any exposure were coded as 0. This lead to a total of 18 timing variables. Duration was defined as the total number of years of experienced CM and calculated by summing up the timing variables leading to a score ranging from 0 to 18.

#### Frequency and Subjective Severity

Frequency and severity were allocated using the maximum answer scores of the follow-up questions. Frequency was assessed by asking how often did this happen to the participant, with answer categories too many times to count, between 10 and 50 times, and fewer than 10 times. Severity was assessed as how did this experience hurt or harm the participant, with answer categories a great deal, seriously, mildly, or not at all. Answers were inverted for scores, so that higher score indicated more frequency or severity.

#### Physical Multimorbidity

Individuals were asked if they experience the following common diseases in the Western world: obesity, diabetes, cancer, hypertension, myocardial infarction, COPD, and incident stroke and answered no or yes. A sum score of these self-reported diseases was built and ranged from 0 to 7.

#### Control Variables

Current age of the respondents as well as their monthly net household income in euros were used as control variables. These factors were assessed because are known to be associated with somatic diseases.

### Statistical Analysis

Characteristics of CM were compared between women and men applying χ^2^ tests (categorical variables) and *t* test (continuous variables). Due to substantial multicollinearity, associations of characteristics of CM with physical multimorbidity in adulthood cannot be tested using conventional analytical techniques. Therefore, we applied conditioned random forest regression, which is a machine learning–based regression that has been shown to achieve a high estimation accuracy in studies where a large amount of data is generated for each individual and provide descriptive variable importance measures reflecting the impact of each variable in associations with the dependent variable.^30^ The importance of each variable is assessed by randomly permuting each variable in turn and determining how much this degrades model fit.^31^ This technique does not require variables to be normally distributed and is not susceptible to multicollinearity.^31^

The mean value of importance or variance of the risk factor variables was calculated with the cforest package in R statistical software version 4.2.1 (R Project for Statistical Computing) random forest with conditional trees to provide an unbiased estimate of variable importance that is not influenced by number of categories.^30^ Training and testing were accomplished using 10 repetitions of 10-fold cross validation. We calculated the mean estimation validity and variable importance measures across the 10-fold cross validation and reliability was derived from the 10 random split repetitions. We included variables for age and maltreatment type in the models to indicate the maximal importance of exposure to each type of maltreatment, regardless of age, and maximum sensitivity to maltreatment at each age regardless of type of exposure. Multiplicity, duration, frequency, and severity were estimated in their importance for physical multimorbidity. A high and significant importance score implies that a specific variable was more importantly associated with the outcome than other variables in the model and remains associated when all other variables in the model are considered. To statistically validate the variable importance scores, random forest regression analysis was performed 5000 times, each time reshuffling the outcome variable to simulate random chance and recalculating the variable importance for each risk factor. This approach allowed us to build a distribution of chance-based importance scores, against which we could compare the observed variable importance scores for statistical significance. The estimated power of type and timing against the estimated strength of multiplicity, duration, and severity was tested.

Since the prevalence of physical diseases tends to increase with age, sensitivity analyses were performed using multiple linear regression analyses with interaction terms between multiplicity, duration, and severity with age groups based on the respondent’s current age to examine if and how the association between CM and physical multimorbidity varied across different age cohorts.

*P* values were 2-sided, and statistical significance was set at *P* < .05. Analyses took place between June 2023 and July 2024.

## Results

### Characteristics of CM

The total study sample included 2514 participants (mean [SD] age, 50.1 [18.0] years) with a mean (SD) monthly net household income of €2782 (€1603 [; US $2891 [$1666]). A total of 674 individuals (26.8%) had a lower educational level (ie, practical apprenticeship entrance qualification degree), 1159 individuals (46.2%) had an intermediate education level (ie, higher qualified apprenticeship [practical and administrative] entrance qualification degree), and 579 individuals (23.0%) had completed higher education (ie, university entrance qualification degree); 1045 persons (41.6%) were married, and 1421 persons (56.7%) were employed full- or part-time. The study included 1297 women (51.6%) (mean [SD] age, 50.6 [17.9] years) and 1217 men (48.4%) (mean (SD) age, 49.5 [18.2] years) (Table 1). Descriptive analyses found mean (SD) multiplicity of 0.69 (1.09) for women and 0.71 (1.08) for men. Exposure to at least 1 subtype of CM was reported by 474 women (36.5%) and 444 men (36.5%). More women (41 [3.2%]) than men (23 [1.9%]) experienced all 4 subtypes of CM (χ^2^ = 4.090; *P* = .04). Physical abuse was significantly more commonly reported by men, while sexual abuse was more commonly reported by women. Men had experienced CM more often at ages 8, 9, and 12 years; women experienced CM more often at age 17 years. Duration, frequency, and severity of CM did not significantly differ between women and men (Table 1).

**Table 1.** Sample Characteristics Stratified for Women and Men

Characteristic	Individuals, No. (%)		*P* value
	Women (n = 1297)	Men (n = 1217)	
Age, mean (SD), y	50.6 (17.9)	49.5 (18.2)	.14
Household income, mean (SD), €	2672 (1531)	2898 (1670)	<.001
CM subtype			
Neglect	202 (15.6)	217 (17.8)	.13
Physical abuse	238 (18.4)	295 (24.2)	<.001
Emotional abuse	296 (22.8)	280 (23.0)	.91
Sexual abuse	164 (12.6)	73 (6.0)	<.001
Multiplicity (ie, sum score subtypes CM), mean (SD)	0.69 (1.09)	0.71 (1.08)	.70
Age of CM, y			
1	8 (0.6)	4 (0.3)^a^	.30
2	10 (0.8)	8 (0.7)	.74
3	19 (1.5)	18 (1.5)	.98
4	40 (3.1)	32 (2.6)	.50
5	67 (5.2)	57 (4.7)	.58
6	101 (7.8)	106 (8.7)	.40
7	128 (9.9)	127 (10.4)	.64
8	147 (11.3)	171 (14.1)	.04
9	158 (12.2)	186 (15.3)	.02
10	203 (15.7)	216 (17.7)	.16
11	192 (14.8)	195 (16.0)	.40
12	203 (15.7)	227 (18.7)	.05
13	190 (14.6)	197 (16.2)	.29
14	189 (14.6)	180 (14.8)	.88
15	180 (13.9)	167 (13.7)	.91
16	167 (12.9)	138 (11.3)	.24
17	135 (10.4)	92 (7.6)	.01
18	85 (6.6)	74 (6.1)	.63
Duration (ie, sum score years experienced CM), mean (SD), y	1.71 (3.29)^b^	1.80 (3.40)^b^	.50
Frequency, mean (SD)	0.56 (0.91)	0.57 (0.92)	.86
Severity, mean (SD)	0.79 (1.23)	0.73 (1.14)	.18
Physical multimorbidity,	0.60 (0.93)	0.64 (1.04)	.35
Mean (SD), No.			
Overweight	263 (20.3)	206 (16.9)	.03
Diabetes	103 (7.9)	114 (9.4)	.20
Cancer	52 (4.0)	34 (2.8)	.10
Hypertension	304 (23.4)	316 (26.0)	.15
Myocardial infarction	26 (2.0)	56 (4.6)	<.001
Chronic obstructive pulmonary disease	20 (1.5)	25 (2.1)	.33
Stroke	18 (1.4)	37 (3.0)	.01

Abbreviation: CM, child maltreatment.

^a^ Due to an insufficient amount of men who experienced child maltreatment at age 1, this result is not reliable and should not be interpreted.

^b^ Mean values of duration were calculated across the complete sample. Because of our representative sample, many study participants did not experience CM and were attributed value 0 for duration. Therefore, mean values of duration cannot be compared with values from patient-based samples.

### Physical Multimorbidity and CM Multiplicity

Women had a mean (SD) physical multimorbidity score of 0.60 (0.93), and men had a mean (SD) score of 0.64 (1.04). Diagnosis of at least 1 physical disease was reported by 491 women (37.9%) vs 436 men (35.8%).

**Figure 1.**
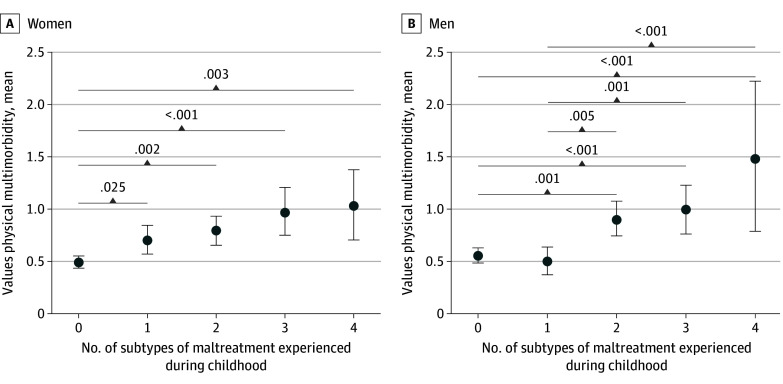
Values of Physical Multimorbidity Stratified for Number of Subtypes of Experienced Child Maltreatment Error bars indicate 95% CIs.

For both women and men, physical multimorbidity was positively correlated with multiplicity (women: *r* = 0.174; *P* < .001; men: *r* = 0.172; *P* < .001). For women, exposure to at least 1 subtype of CM increased the mean value of physical multimorbidity in adulthood, whereas for men this was found for 2 or more subtypes of CM. Having experienced all 4 subtypes of CM was associated with higher mean values of physical multimorbidity in men compared with women (Figure 1).

### Conditioned Random Forest Regression Analyses

In women, duration of CM was found to be the most important factor associated with physical multimorbidity in adulthood (importance = 0.595; 95% CI, 0.599-0.601). Subjective severity and experienced CM at age 4 years had significant importance for physical multimorbidity in adulthood as well as control variables age and income. When the power of type and timing were tested against the strength of global scores (ie, multiplicity, duration, and severity), duration, severity, and timing of CM (exposure at ages 4, 10, 11, 14, and 17 years) showed more important associations with physical multimorbidity than multiplicity (Table 2).

**Table 2.** Importance of Risk Factors for Physical Multimorbidity in Women and Men

Factor	Importance (SD)	*P* value	Tested against, importance		
			Multiplicity	Duration	Severity
**Women**					
Neglect	0.08 (0.005)	.43	−7.72	−15.19	−23.42
Physical abuse	0.23 (0.009)	.21	1.44	−10.74	−11.47
Emotional abuse	0.01 (0.003)	.76	−12.74	−23.61	−23.96
Sexual abuse	−0.03 (0.002)	.75	−14.18	−22.87	−28.21
Multiplicity	0.27 (0.010)	.09	NA	9.18	10.28
CM age, y					
1	0.00 (0.000)	.95	−15.27	−21.19	−28.16
2	0.00 (0.000)	.69	−15.28	−21.20	−28.21
3	0.00 (0.000)	.38	−14.87	−21.13	−27.81
4	0.28 (0.006)	.02	4.32^a^	−9.47	−10.85
5	0.09 (0.003)	.21	−7.34	−16.09	−21.58
6	0.10 (0.003)	.26	−6.62	−15.64	−19.86
7	0.02 (0.003)	.57	−11.62	−19.36	−24.89
8	0.06 (0.003)	.50	−9.60	−17.68	−25.14
9	0.01 (0.003)	.69	−12.57	−20.33	−25.76
10	0.47 (0.014)	.07	11.66^a^	−1.80	−2.19
11	0.44 (0.022)	.07	10.19^a^	−2.26	−2.98
12	0.11 (0.008)	.43	−6.02	−13.92	−18.77
13	0.15 (0.004)	.32	−3.42	−14.62	−17.06
14	0.53 (0.014)	.05	16.47^a^	0.37	0.33
15	0.21 (0.006)	.21	0.71	−10.09	−15.27
16	0.04 (0.003)	.54	−10.52	−18.92	−24.88
17	0.25 (0.005)	.13	3.08^b^	−9.72	−12.11
18	0.22 (0.004)	.11	0.75	−12.53	−13.94
Duration	0.60 (0.027)	.01	13.24^a^	NAs	2.37^c^
Frequency	0.00 (0.008)	.73	−10.32	−19.85	−26.71
Severity	0.63 (0.031)	.02	16.82^a^	3.00^b^	NA
Age at survey participation	19.13 (0.478)	<.001	NA	NA	NA
Income	3.20 (0.075)	<.001	NA	NA	NA
**Men**					
Neglect	0.05 (0.005)	.54	−10.44	−42.11	−8.35
Physical abuse	0.13 (0.007)	.42	−5.47	−32.09	−1.89
Emotional abuse	−0.02 (0.002)	.81	−14.94	−42.54	−15.33
Sexual abuse	0.27 (0.005)	.07		−32.38	7.41^a^
Multiplicity	0.32 (0.017)	.08	1.95^c^	−26.31	7.45^a^
CM age, y					
1	0.00 (0.000)	1.00	−13.34	−43.14	−15.66
2	0.00 (0.000)	.89	−13.32	−43.16	−15.75
3	0 (0.001)	.35	−13.09	−42.65	−14.67
4	0.01 (0.001)	.39	−12.86	−42.73	−13.66
5	0.01 (0.001)	.51	−11.90	−43.23	−13.63
6	0.28 (0.006)	.09	0.45	−31.56	7.27^a^
7	0.1 (0.005)	.35	−6.97	−35.58	−4.36
8	0.03 (0.004)	.61	−12.24	−38.61	−10.75
9	0.07 (0.007)	.51	−8.19	−36.47	−5.23
10	0.06 (0.003)	.55	−9.43	−38.85	−7.30
11	0.7 (0.029)	.03	14.00^a^	−12.58	20.12^a^
12	0.06 (0.003)	.59	−9.15	−39.20	−7.21
13	0.46 (0.016)	.08	7.55^a^	−20.37	15.70^a^
14	0.38 (0.014)	.10	4.33^a^	−24.06	11.63^a^
15	0.19 (0.003)	.25	−3.71	−36.06	2.43^b^
16	0.11 (0.007)	.34	−7.46	−34.65	−3.77
17	0.02 (0.002)	.52	−11.59	−41.58	−9.78
18	0.11 (0.003)	.22	−7.99	−37.92	−4.39
Duration	1.39 (0.065)	<.001	25.15^a^	NA	33.85^a^
Frequency	0.19 (0.011)	.26	−2.92	−32.80	1.85^c^
Severity	0.2 (0.009)	.23	−3.06	−34.22	
Age at survey participation	21.64 (0.93)	<.001	NA	NA	NA
Income	1.51 (0.029)	.001	NA	NA	NA

Abbreviations: CM, child maltreatment; NA, not applicable.

^a^ *P* < .001.

^b^ *P* < .01.

^c^ *P* < .05.

**Figure 2.**
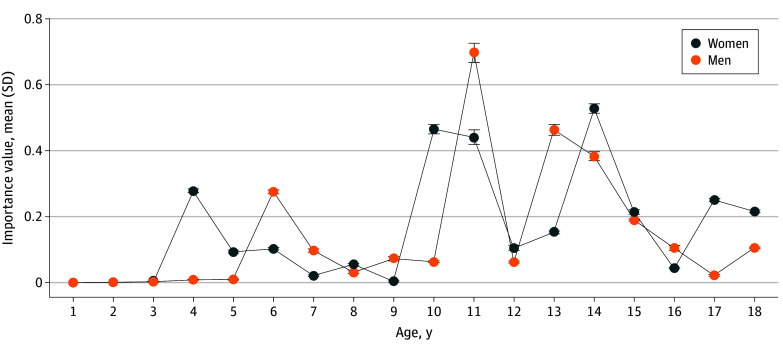
Importance of Timing of Experienced Child Maltreatment for Physical Multimorbidity in Adulthood

In men, duration of CM was found to have the highest strength of association for physical multimorbidity in adulthood (importance = 1.389; 95% CI, 1.386-1.394). Experience of CM at age 11 years showed significant importance for physical multimorbidity in adulthood as well as age and income. Duration and timing of CM (exposure at ages 11, 13, or 14 years) revealed more important associations for physical multimorbidity than multiplicity. Duration, experience of sexual abuse, and timing of CM (exposure at ages 6, 11, 13, 14, or 15 years) was more importantly associated with physical multimorbidity than severity (Table 2). The age course of experienced CM and its strength of association for physical multimorbidity in adulthood for women and men fluctuated (Figure 2).

### Interactions of Multiplicity, Duration, and Severity With Current Age Groups

**Figure 3.**
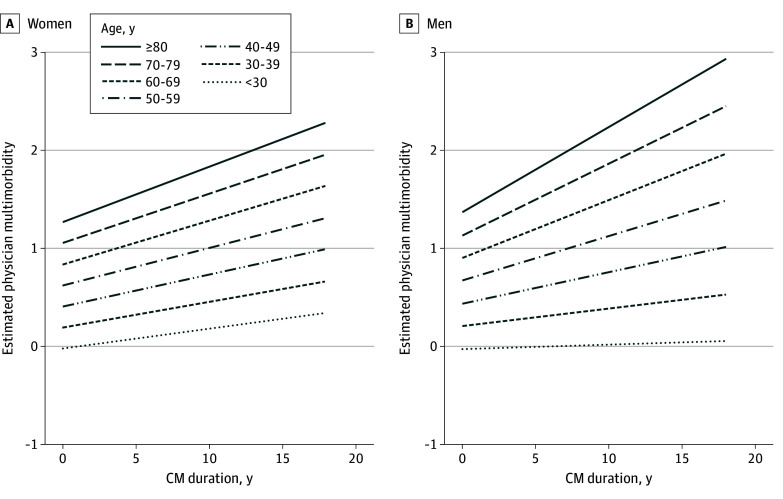
Estimated Physical Multimorbidity by Duration of Child Maltreatment (CM) and Age Groups for Women and Men Included covariates in the model are neglect and physical, emotional, and sexual abuse; frequency and severity of CM. The model explained 22.1% (adjusted *R*^2^ = .221) of variance for women and 23.4% (adjusted *R*^2^ = .234) of variance for men.

A significant interaction effect for duration of CM with age was found for men (β = 0.264; 95% CI, 0.001-0.002; *P* = .001). With higher number of years of experienced CM, physical multimorbidity increased more for older age groups compared with younger age groups (Figure 3). For women, no significant interaction between duration of CM and age was found (β = 0.133; 95% CI, 0.000-0.001; *P* = .09). Interactions for multiplicity and severity with age are presented in eFigure 1 and eFigure 2 in Supplement 1.

## Discussion

To our knowledge, this survey study is the first study examining numerous characteristics of CM (type, timing, duration, severity, and frequency) in their associations with physical multimorbidity in adulthood using a large representative sample. Using dose-dependent risk assessment of multiplicity for physical multimorbidity, we found overall duration and timing of CM to be more imporant risk factors associated with physical multimorbidity than multiplicity in both women and men. Over and above research using crude aggregate measures of CM, our work highlights the necessity to have a more nuanced and sophisticated approach of measuring and modeling the impacts of CM.

The significant association that we found for overall duration of CM and multimorbidity is in accordance with a 2001 study by Thornberry et al^32^ that found persistent maltreatment to have stronger and more negative associations with health outcomes than maltreatment only experienced in 1 developmental stage. Furthermore, repeated exposure (ie, exposure across multiple age categories) was associated with increased risk for problematic cardiometabolic health (eg, obesity and diabetes).^33^

Important mediators between CM and long-term health encompass a dysregulation of the hypothalamic-pituitary-adrenal axis and the autonomic nervous system as a response to enormous stress caused by CM, producing physiological changes that are maladaptive in the long term^34^ and linked to adverse health outcomes, such as cardiovascular and metabolic diseases.^6^ Since alterations in the brain are strongest when induced by repeated or chronic stress,^35^ a longer duration of exposure to CM increases the likelihood of brain alterations and thus the likelihood of physical multimorbidity. CM is associated with chronic low-grade inflammation in adulthood,^36,37^ a key factor in cardiovascular disease,^38^ cancer,^39^ diabetes, and metabolic diseases.^40^

Timing aspects showed associations with physical multimorbidity. Early experience of CM (age 4 years) for women and prepuberty CM (age 11 years) for men were associated with physical multimorbidity. CM causes alterations in the biological system,^8^ ie, it influences trajectories of a child’s brain development,^26^ which depends on the timing and type of exposure.^26^ Brain structure differs between boys and girls,^41^ ie, boys have greater brain structure variability than girls.^41^ Major changes in the brain occur in early childhood and puberty, especially vulnerable phases in which children are more susceptible to negative outcomes associated with CM.^26^ Furthermore, women and men differ in metabolic, psychological, and lifestyle-related risk factors for chronic diseases.^42^

Age influenced the association between duration of CM and physical multimorbidity in men: older age groups had greater physical multimorbidity compared with younger age groups. Middle-aged men display an increased number of medical conditions after adverse childhood experiences,^43^ whereas associations between exposure to abuse and internalizing mental disorders (eg, depression) are stronger for women.^44^ Furthermore, a negative socioemotional adjustment after having experienced CM can result in men being less likely to maintain satisfying social relationships that may have a protective association against adverse stress outcomes involving traumatic events;^45^ this negative socioemotional adjustment has been associated with increased health risk behaviors,^46^ especially substance use and abuse in adolescence^47^ and beyond.^48^ Engagement in health care is also less common among men compared with women^49^ and lower among individuals who were exposed to adverse childhood experiences,^50^ which could contribute to adverse health outcomes in adulthood.

### Strengths and Limitations

This study has some strengths. The major advantage of this study is the representativeness of the sample, ensuring a high generalizability of the results. Our study is, to our knowledge, the first to collect in-depth data on subtypes, timing, duration, frequency, and severity of CM in a representative, nationwide sample. The assessment of the specific ages at which each subtype of CM was experienced and the use of conditioned random forest regression analyses enabled us to determine the most important aspects of CM in estimating somatic outcomes in adulthood.

This study also has some limitations. A major limitation is the retrospective assessment of CM. There has been a critical debate on the validity of retrospectively assessed CM^51^; however, subjective reports of CM were shown to be highly relevant for adulthood.^52^ Due to the impaired understanding and remembrance of events happening in early childhood, our data assessing CM in very young ages must be seen with caution. Longitudinal assessments of CM in cohorts starting with birth are needed to give insight into the prevalence of CM in this highly sensitive period. In our study, information about social determinants of health during childhood is lacking, and potentially confounds the association between CM and adult health outcomes. Furthermore, the measure of physical multimorbidity was self-reported, which could affect our results. However, agreement between self-report and medical record for diabetes, hypertension, myocardial infarction, and stroke has been shown to be substantial.^53^ Also the use of our multimorbidity score has its limitations. Although it offers valuable insights into overall physical health, the impact of CM on specific health conditions may vary, and a more granular approach focusing on each disease could reveal disease-specific patterns of CM.

## Conclusions

This survey study found that a higher duration of CM was the most important characteristic of CM associated with physical multimorbidity in adulthood in both women and men and that timing variables were of further importance. Both duration and timing had more important associations with physical multimorbidity than multiplicity of CM exposure. Our findings call for consideration of CM assessments in diagnostics of individuals with physical health conditions. Awareness of these risk factors may enable us to identify individuals at greatest risk, modify treatment recommendations accordingly, and inform prevention and policy. Future research should particularly focus on the vulnerability in specific age periods and investigate underlying mechanisms that may increase subsequent risks. Focusing on societal implications, there is an urgent need to prevent CM or, if happening, to stop any form of CM as soon as possible to decrease the major public health burden related to the long-term health outcomes associated with CM, and, most importantly, the individual burden of each affected human.

## Notes

Supplement 1. **eFigure 1.** Estimated Physical Multimorbidity by Multiplicity of CM and Age Groups for Women and Men **eFigure 2.** Estimated Physical Multimorbidity by Severity of CM and Age Groups for Women and Men

Supplement 2.
Data Sharing Statement
