# Supplementary material for: Child Maltreatment Characteristics and Adult Physical Multimorbidity in Germany
Source: JAMA Netw Open. 2025 Jan 23;8(1):e2456050. doi: 10.1001/jamanetworkopen.2024.56050 (PMC11759131; doi:10.1001/jamanetworkopen.2024.56050)
Supplement: Supplement 1. — eFigure 1. Estimated Physical Multimorbidity by Multiplicity of CM and Age Groups for Women and Men eFigure 2. Estimated Physical Multimorbidity by Severity of CM and Age Groups for Women and Men [file jamanetwopen-e2456050-s001.pdf]

Supplemental Online Content

Otten D, Schalinski I, Fegert JM, et al. Child maltreatment characteristics and adult physical multimorbidity in Germany. *JAMA Netw Open*. 2025;8(1):e2456050. doi:10.1001/jamanetworkopen.2024.56050

eFigure 1. Estimated Physical Multimorbidity by Multiplicity of CM and Age Groups for Women and Men

eFigure 2. Estimated Physical Multimorbidity by Severity of CM and Age Groups for Women and Men

This supplemental material has been provided by the authors to give readers additional information about their work.

© 2025 Otten D et al. *JAMA Network Open*.

Figure e1. Estimated Physical Multimorbidity by Multiplicity of CM and Age Groups for Women and Men.

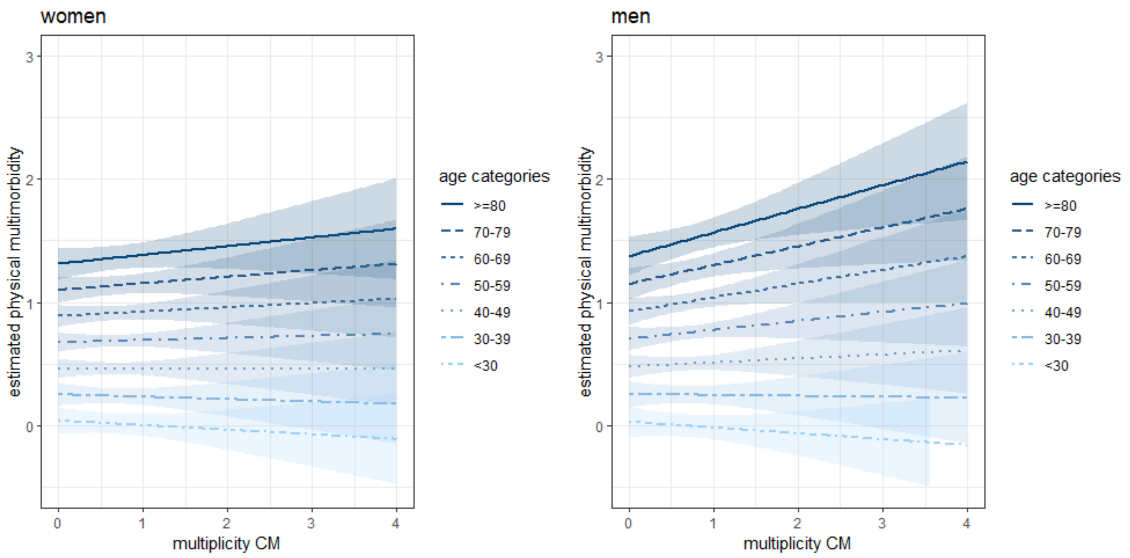

Note: CM = child maltreatment.

Figure description: A significant interaction effect for multiplicity with age was found in the model for men (β = 0.229, 95% CI [0.001 – 0.006], *p* = .002, adjusted R^2 model = .226). With an increase in the number of experienced subtypes of CM, physical multimorbidity increased more strongly for older age groups compared to younger age groups. An interaction effect between multiplicity and age was not found for women (β = 0.096, , 95% CI [-0.001 – 0.004], *p* = .203, adjusted R^2 model = .221). Multiplicity and age do not interact in their influence on physical multimorbidity in women. Included covariates in the models were: duration, frequency, and subjective severity of CM.

© 2025 Otten D et al. *JAMA Network Open*.

Figure e2. Estimated Physical Multimorbidity by Severity of CM and Age Groups for Women and Men.

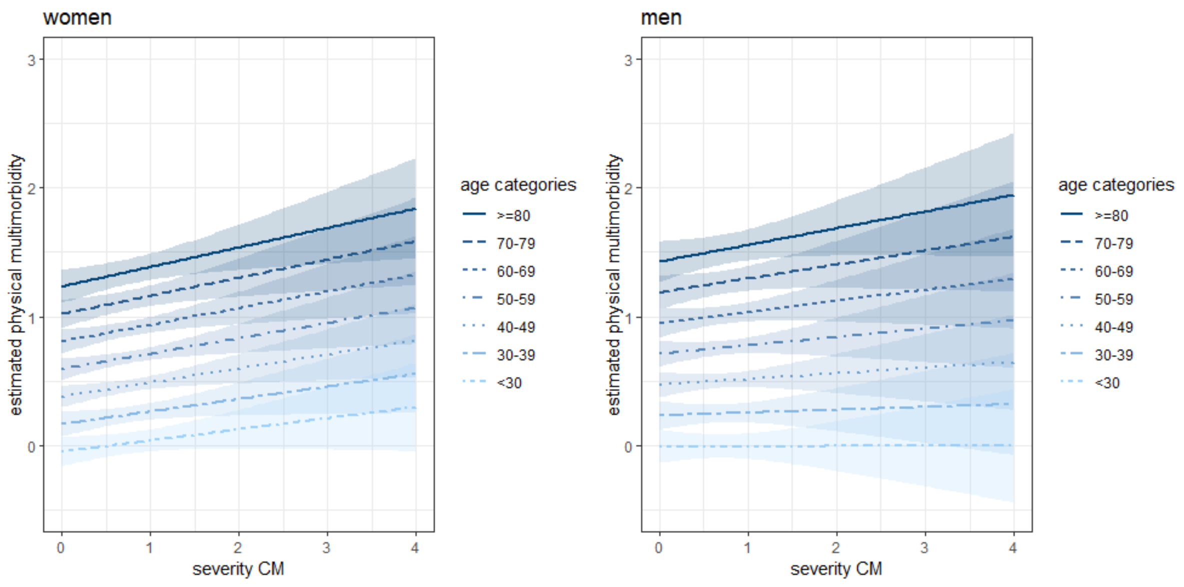

Note: CM = child maltreatment.

Figure description: A significant interaction effect for severity with age was neither found for men (β = 0.137, 95% CI [0.000 – 0.005], *p* = .095, adjusted R^2 model = .231) nor for women (β = 0.059, 95% CI [-0.001 – 0.003], *p* = .462, adjusted R^2 model = .0.225). Severity of CM and age do not interact in their influence on physical multimorbidity in either men or women. Included covariates in the models were: neglect and physical, emotional, and sexual abuse, frequency and timing of CM.

© 2025 Otten D et al. *JAMA Network Open*.
