# Supplementary material for: Child Maltreatment Characteristics and Adult Physical Multimorbidity in Germany
Source: JAMA Netw Open. 2025 Jan 23;8(1):e2456050. doi: 10.1001/jamanetworkopen.2024.56050 (PMC11759131; doi:10.1001/jamanetworkopen.2024.56050)
Supplement: Supplement 2. — Data Sharing Statement [file jamanetwopen-e2456050-s002.pdf]

Data Sharing Statement

Data
Data available: No

Additional Information
Explanation for why data not available: Data are available upon request.
